# FlgM as a Secretion Moiety for the Development of an Inducible Type III Secretion System

**DOI:** 10.1371/journal.pone.0059034

**Published:** 2013-03-12

**Authors:** Thomas Heel, Georg F. Vogel, Andrea Lammirato, Rainer Schneider, Bernhard Auer

**Affiliations:** 1 Institute of Biochemistry, University of Innsbruck, Innsbruck, Austria; 2 Austrian Centre of Industrial Biotechnology, Graz, Austria; 3 Center for Molecular Biosciences (CMBI), University of Innsbruck, Innsbruck, Austria; 4 Division of Histology and Embryology, Medical University of Innsbruck, Innsbruck, Austria; 5 Division of Cell Biology, Medical University of Innsbruck, Innsbruck, Austria; Universidad Nacional Autónoma de México, Mexico

## Abstract

Regulation and assembly of the flagellar type III secretion system is one of the most investigated and best understood regulational cascades in molecular biology. Depending on the host organism, flagellar morphogenesis requires the interplay of more than 50 genes. Direct secretion of heterologous proteins to the supernatant is appealing due to protection against cellular proteases and simplified downstream processing. As *Escherichia coli* currently remains the predominant host organism used for recombinant prokaryotic protein expression, the generation of a strain that exhibits inducible flagellar secretion would be highly desirable for biotechnological applications.

Here, we report the first engineered *Escherichia coli* mutant strain featuring flagellar morphogenesis upon addition of an external inducer. Using FlgM as a sensor for direct secretion in combination with this novel strain may represent a potent tool for significant improvements in future engineering of an inducible type III secretion for heterologous proteins.

## Introduction

In bacteria, motility is mediated through a complex, macromolecular machinery referred to as the flagellar type III secretion system. The assembly of this structure represents a huge burden for the cell and is therefore tightly regulated [1]–[3]. More than 50 genes, classified into three hierarchical groups, are involved in the pathway and regulation thereof [4], [5]. In response to environmental signals, bacterial cells form a functional flagellum, which is initiated by expression of the early class I master operon *flhDC* [6], [7]. It was shown that gene deletion strains lacking the master operon are non-motile [6]. FlhD_4_C_2_ heterohexamers subsequently bind to upstream regulator elements of class II genes resulting in the expression of the respective genes and ultimately in the assembly of the hook basal body [5]. Within the group of class II gene products, the FlgM protein fulfills two important functions. First of all, it binds to FliA, thereby inhibiting premature expression of the late class III filament genes [8], [9]. Secondly, after completed assembly of the hook basal body spanning both cellular membranes, the sensor protein FlgM is secreted into the supernatant using this structure [10]–[12]. These processes result in the release of the late transcription factor FliA and consequently in the expression of the class III filament genes along with complete assembly of the flagellar structure [13], [14].

Using the hook basal body as a channel to directly secrete heterologous proteins would be very appealing for several reasons, such as protection from cellular proteases [15], high purity and as a consequence simplified downstream processing [16], [17]. Depending on the organism investigated, different secretion signals have been proposed to mediate the transport of also of heterologous proteins through the type III machinery [18]–[21]. In contrast to *S. enterica,* there is no available *E. coli* mutant strain up to date, featuring an inducible flagellar secretion system, making systematic investigations regarding this topic more challenging. [14], [22]–[24] Therefore, an *E. coli* strain exhibiting a genomically introduced promoter to induce the type III machinery remains highly desirable.

A recent study has successfully shown that the sensor protein FlgM can mediate secretion of fusion peptides in the related *Salmonella enterica serovar Typhimurium* strain [15]. However, due to the pathogenicity of this model organism [25], [26], we were especially interested in working with the biotechnologically relevant host strain HMS174(DE3) to investigate type III secretion.

In this study, we utilized the sensor function of FlgM along with its subsequent transport across the flagellar hook basal body to isolate the first *E. coli* mutant strain exhibiting inducible secretion of FlgM to the supernatant. More detailed investigations of the respective strain using Scanning Electron Microscopy revealed full flagellar morphogenesis upon addition of an external inducer. Furthermore, we could show that FlgM is not only a useful tool to estimate the impact of host strain modifications on an inducible type III secretion system but could also potentially facilitate recombinant protein secretion itself.

## Results

**Figure 1:**
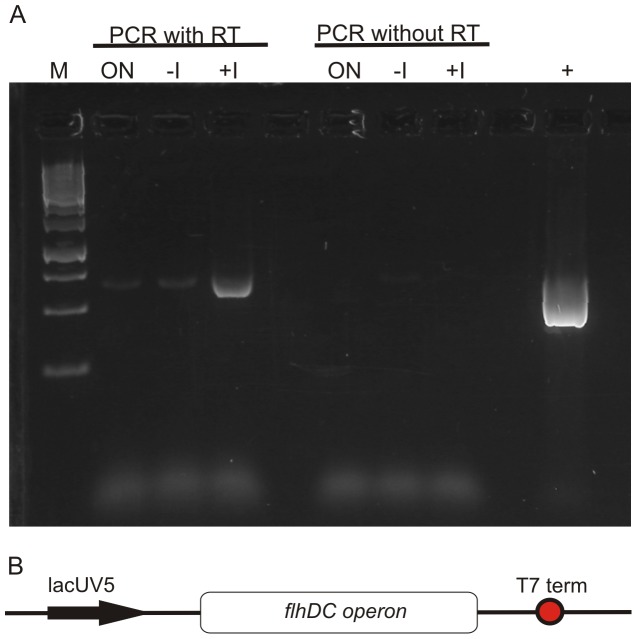
Transcription of the master operon *flhDC*. (**A**) To verify the transcription of the plasmid-encoded master operon *flhDC* Reverse Transcriptase PCR Reaction of the isolated RNA was performed. It was assumed that within the overnight culture cells exhibiting flagellar assembly were found and therefore this culture was used as a positive control. Cells before addition of the inducer IPTG were used as reference compared to cells after induction of the plasmid-encoded *flhDC*. To evaluate genomic DNA contamination the isolated RNA samples were also subjected to subsequent PCR. Plasmid-encoded *flhDC* was used as a positive control for the PCR reaction. Fermentas 1 KB GeneRuler, ON overnight culture sample, -I whole cell sample without induction of plasmid-encoded *flhDC*, +I whole cell samples with induction of plasmid-encoded *flhDC*, RT Reverse Transcriptase Reaction, + plasmid-encoded *flhDC*; (**B**) Scheme of the plasmid-encoded master operon under the control of the lacUV5 promoter

**Figure 2:**
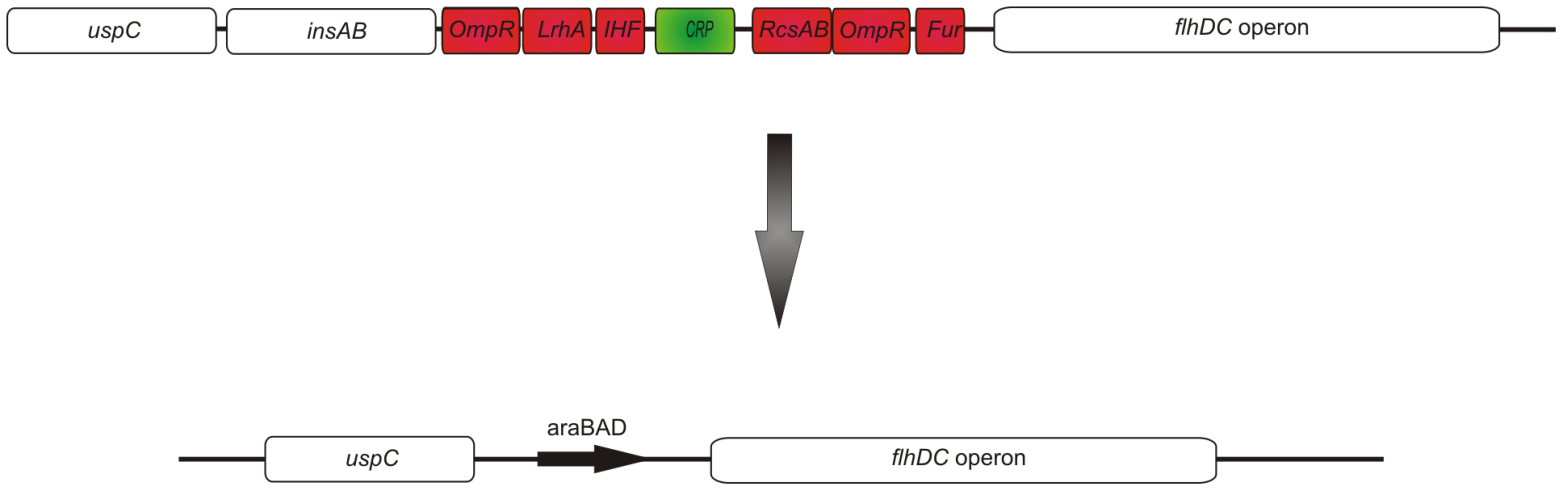
Modification of the *flhDC* master operon. The expression strain HMS174(DE3) was used for modifications of the bacterial genome. Upstream regulator elements of the master operon *flhDC* were deleted and replaced by the artificial araBAD promoter resulting in the HMS174(DE3)Δ*insAB* araBAD-*flhDC* strain. Activator elements are depicted in green, whereas repressor elements are depicted in red [6], [40].

Initial experiments were performed using plasmid-encoded overexpression of the master operon *flhDC* to induce flagellar assembly in the *E. coli* strain HMS174(DE3). Reverse Transcriptase PCR experiments to confirm plasmid-encoded transcription of the master operon *flhDC* revealed a significant disadvantage of this approach as basal transcription levels of the master operon were detected (Figure 1). This could either be the result of plasmid-encoded promoter leakage or due to basal transcription levels of the genomic master operon in a major fraction of the analyzed samples. Therefore, higher homogeneity of the *E. coli* cultures regarding initiation of the flagellar machinery was desirable for more detailed investigations. In theory, this should be achievable by integration of an artificial promoter upstream of the flagellar master operon. Since RT-PCR experiments of *flhDC* transcripts do not ensure actual initiation of the flagellar regulation cascade and morphogenesis, we additionally pursued a different approach. The master regulator FlhD_4_C_2_ leads to the expression of class II proteins necessary for the assembly of the hook basal body including the sensor protein FlgM [5], [6]. Given that FlgM is secreted to the supernatant via this structure after its functional assembly, the protein can only be found in the supernatant if the expression of the master regulator *flhDC* was sufficient to induce flagellar morphogenesis in the majority of the cultivated cells [11], [13], [27]. We took advantage of this circumstance to perform the following functional assay, recombinant expression of His-tagged FlgM protein to screen genetically modified host strains. This enabled systematic investigations of the impact of gene modifications on the development of a new *E. coli* host strain efficiently secreting FlgM upon addition of an external inducer. Previous studies have reported successful *flhDC* expression from a genomically introduced Tetracycline promoter as well as with plasmid-encoded Arabinose promoter in *S. enterica serotype Typhimurium* [24], [28], [29]. In a first step, this strategy was conveyed to *E. coli*. Since several global regulators influence the expression of the master operon [22], [30], we replaced all upstream elements with the araBAD promoter (Figure 2), resulting in the *E. coli* HMS174(DE3)*ΔinsAB* araBAD-*flhDC* strain.

**Figure 3:**
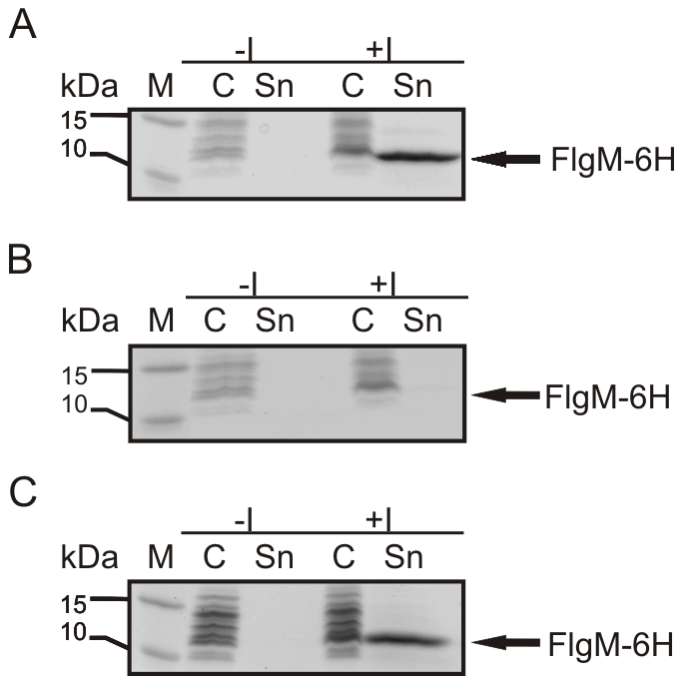
Comparison of the secretion competency via plasmid-encoded overexpression of FlgM. (A) FlgM was secreted to the supernatant in the HMS174(DE3) strain. **(B)** In contrast, in the newly generated HMS174(DE3)Δ*insAB* araBAD-*flhDC* no FlgM could be detected in the supernatant. **(C)** The ability to secrete FlgM was recovered in the HMS174(DE3)Δ*insAB* araBAD-*flhDC* strain when plasmid-encoded *flhDC* was co-expressed. SDS-PAGE, M Fermentas PageRuler Prestained, -I whole cell sample without induction of recombinant protein expression, +I whole cell samples with induction of recombinant protein expression, C cytoplasmic fraction, Sn supernatant;

**Figure 4:**
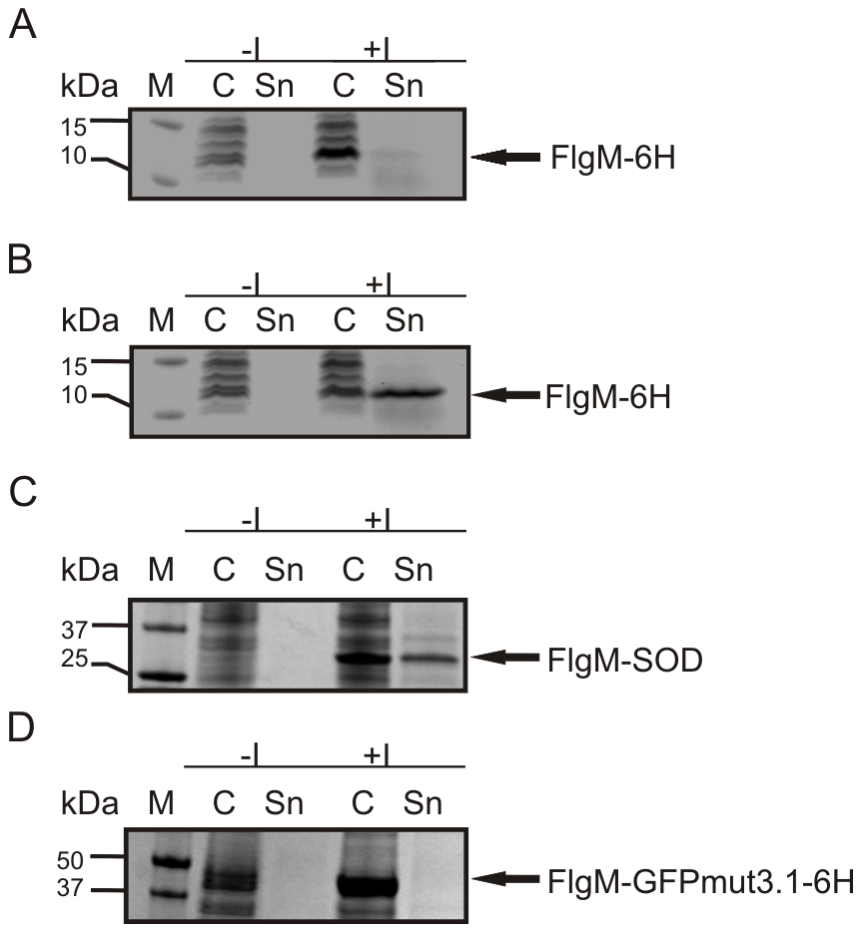
Inducible secretion of FlgM. In the newly generated strain HMS174(DE3)LT7 the functional assembly of the hook basal body led to the secretion of plasmid-encoded FlgM into the supernatant. No secretion of FlgM occurred in the strain HMS174(DE3)LL. **(A)**, HMS174(DE3)LL, pET30-lacUV5-*flgM-6His* **(B)**, HMS174(DE3)LT7, pET30-lacUV5-*flgM-6His* **(C)** HMS174(DE3)LT7, pET30-lacUV5-*flgM-SOD* **(D)** HMS(174)LT7, pET30-lacUV5-*flgM-GFPmut3.1-6H*, M Fermentas PageRuler Prestained, -I whole cell sample without induction of recombinant protein expression, +I whole cell samples with induction of recombinant protein expression, C cytoplasmic fraction, Sn supernatant;

Since we could not detect FlgM in the supernatant we assumed that the feedback inhibitor FliT in the respective strain HMS174(DE3)*ΔinsAB* araBAD-*flhDC* inhibited the function of the heterohexamer FlhD_4_C_2_ [31]. However, deletion of the *fliT* gene did not lead to detectable secretion of the FlgM protein to the supernatant (Figure S1). Plasmid-encoded expression of the master operon under the control of the lacUV5 promoter however restored the ability of the strain to secrete recombinantly expressed FlgM (Figure 3). This indicated that the Arabinose promoter did not give rise to sufficient transcription levels of the *flhDC* operon in order to induce hook basal body assembly in *E. coli*. In subsequent experiments we modified the transcription levels of the master operon using different promoters, such as lacUV5 and T7. However, none of these modifications led to detectable secretion of FlgM (Figure S1). These results led us to the conclusion that transcription levels obtained with a single copy of *flhDC* under the control of the lacUV5, araBAD or the T7 promoter, respectively, were not sufficient to trigger the expression of the class II genes in *E. coli*. Consequently, we introduced a second copy of the master operon under the control of the lacUV5 promoter into the *E. coli* genome at the well-characterized att7 integration site [32], [33]. The resulting strain combining two copies of *flhDC,* HMS174(DE3)*ΔinsAB*, lacUV5-*flhDC*, att7 lacUV5-*flhDC*, further referred to as HMS174(DE3)LL, did not show FlgM secretion either (Figure 4). Therefore, the strong T7 promoter was integrated in exchange for the lacUV5 promoter at the att7 transposon site, resulting in the HMS174(DE3)*ΔinsAB*, lacUV5-*flhDC*, att7 T7-*flhDC* strain, further referred to as HMS174(DE3)LT7. Finally using this strain, plasmid-encoded FlgM was successfully secreted via the flagellar hook basal body (Figure 4).

As we observed the secretion of a linker-6His tag comprised of 15 amino acids to the supernatant when fused to FlgM with high purity we were interested to determine if larger fusion polypeptides would also be efficiently secreted. Subsequent experiments with human Superoxide Dismutase fused to FlgM revealed the potential of FlgM to serve as a secretion moiety for fusion peptides exhibiting higher molecular weight. However, GFPmut3.1-6His protein could not be found in the supernatant when fused to FlgM, which may be due to premature folding of GFP in the cytoplasm (Figure 4, Figure 4S).

### Characterization of the HMS174(DE3)LT7 strain

As this particular strain exhibited inducible secretion of FlgM we were interested in a more detailed characterization of this modified strain.

**Figure 5:**
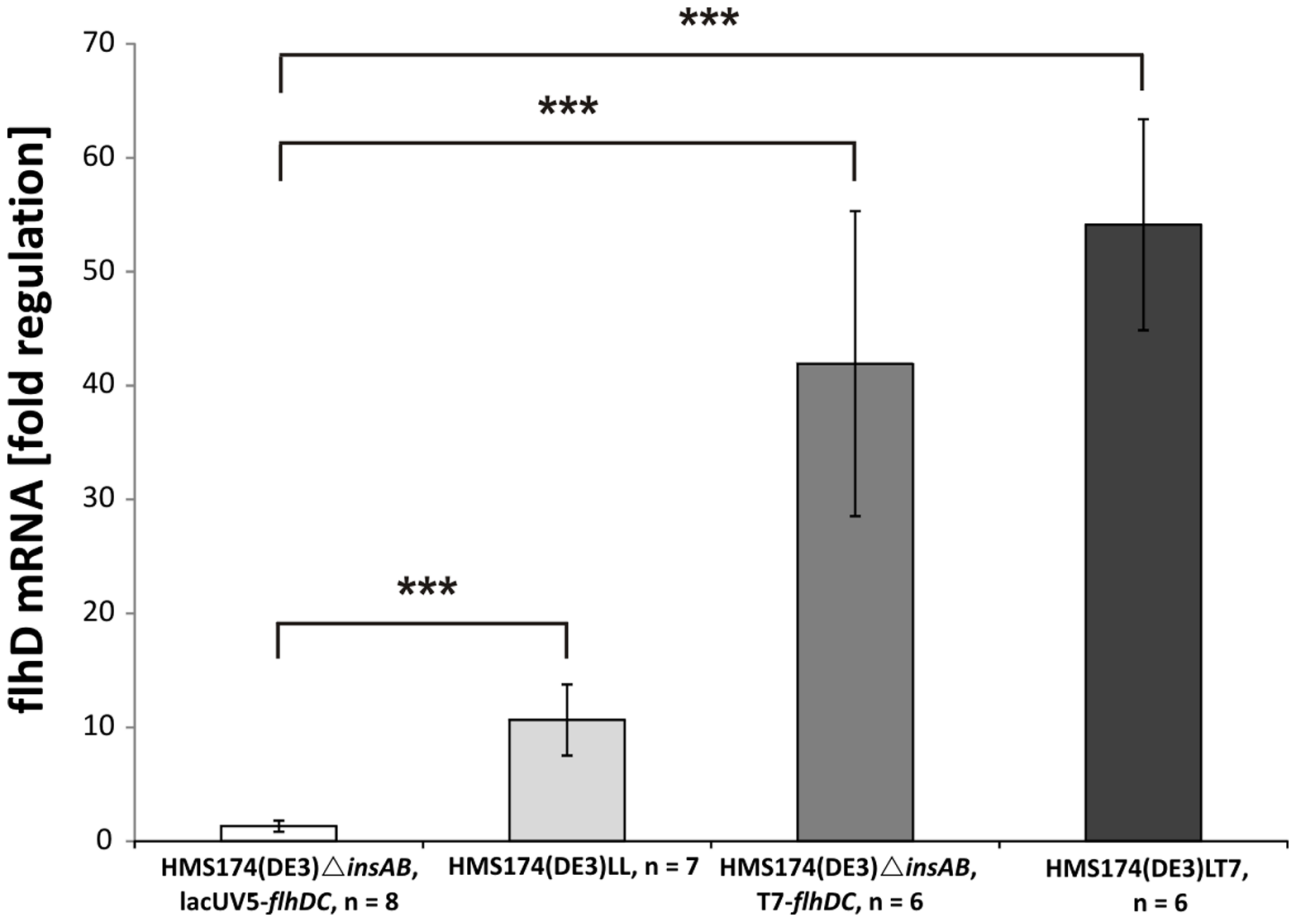
Quantitative Real Time PCR of generated HMS174(DE3) mutant strains. (A) The generated mutant strains HMS174(DE3)Δ*insAB* lacUV5-*flhDC*, HMS174(DE3)Δ*insAB* lacUV5-*flhDC*, att7 lacUV5-*flhDC*, HMS174(DE3) Δ*insAB* T7-*flhDC* and HMS174(DE3)LT7 were incubated for 1 h after induction with 1 mM IPTG at 37°C/225 rpm. Quantification of flhD mRNA levels was accomplished with the comparative ΔΔCt method using rpoD for normalization. Resulting data were analyzed via 2-tailed type 2 Student's *t*-test, ***p<0.001, n = number of replicates.

It was shown that FlgM has a negative impact on class II gene expression via binding of FliA [34]. Hence, we assumed that a distinct threshold of FlhD_4_C_2_ is necessary to overcome the effect of plasmid-encoded FlgM expression. To support this hypothesis quantitative Real-Time (qRT) PCR analyses of the host strains HMS174(DE3)*ΔinsAB*, lacUV5-*flhDC,* HMS174(DE3)*ΔinsAB*,T7-*flhDC,* HMS174(DE3)LL, HMS174(DE3)LT7 were performed to determine the actual differences of *flhD* mRNA levels. Although HMS174(DE3)*ΔinsAB*, lacUV5-*flhDC* and HMS174(DE3)LL exhibited lower *flhD* mRNA levels, no significant difference was observed in the HMS174(DE3)*ΔinsAB*,T7-*flhDC* or HMS174(DE3)LT7 strains (Figure 5). This was rather unexpected considering inducible secretion of FlgM could only be observed in HMS174(DE3)LT7 (Figure 4, Figure S1). This indicated that two gene copies of the master operon with different promoter strengths might result in altered temporal mRNA levels ideal for the secretion of FlgM in shaking flask cultivation.

**Figure 6:**
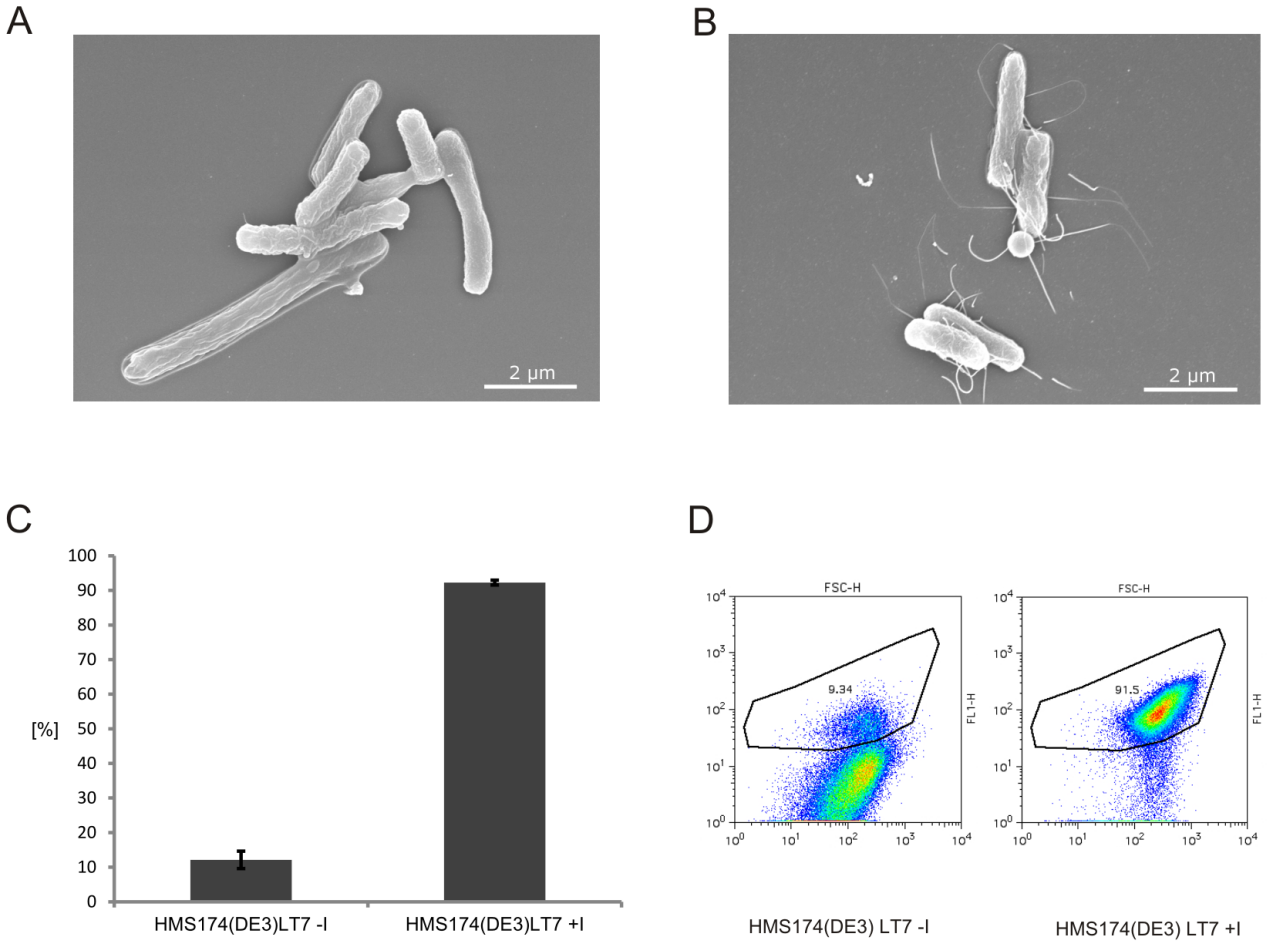
Analysis of the novel host strain HMS174(DE3)LT7. (A) Scanning Electron Microscopy (SEM) of the HMS174(DE3)LT7 strain without addition of the inducer IPTG. **(B)** Scanning Electron Microscopy (SEM) of the HMS174(DE3)LT7 strain with addition of the inducer IPTG. Samples were prepared after 90 min cultivation. **(C)** **Cell culture homogeneity.** The homogeneity regarding *fliC* promoter activity of three parallel HMS174(DE3)LT7 strain cultivations containing the plasmid p5′UTR-f*liC20-GFPmut-Flag-Linker-StrepII*-3′UTR with and without addition of the inducer IPTG were analyzed via Fluorescence Activated Cell Sorting (FACS). After incubation for 2 h at 220 rpm at 37 °C all samples were normalized to OD 600: 1.0 and the percentage of GFP expressing cells indicating flagellar assembly was determined. **(D)** **FACS analysis of GFP expressing cells**. A sample of the HMS174(DE3)LT7 strain cultivation containing the plasmid p5′UTR-*fliC20-GFPmut-Flag-Linker-StrepII*-3′UTR (from Figure 6C) with and without addition of the inducer IPTG.

Since FlgM is only secreted once a functional hook basal body is fully assembled, we were interested to determine whether this mutant strain also assembled flagellar filaments upon addition of the external inducer IPTG. Using Scanning Electron Microscopy of the novel HMS174(DE3)LT7 strain we could confirm complete assembly of the this structure including the final filament upon addition of an external inducer (Figure 6 A, 6B).

The major component of this filament is the late class III protein FliC [35]. To determine the fraction of HMS174(DE3)LT7 cells exhibiting promoter activity of the respective *fliC* gene upon induction of the master operon we performed Fluorescence Activated Cell Sorting (FACS). For this purpose we used a plasmid encoding GFP under the control of the *fliC* promoter [21], [36], [37]. In these experiments HMS174(DE3)LT7 cultivations harboring this construct with or without addition of the inducer IPTG were compared. We could show that the majority of the cells indeed exhibited GFP expression upon addition of the inducer, whereas only a minor fraction of non-induced cells showed considerable GFP levels (6C, 6D). This analysis indicated a high level of homogeneity with respect to the class III promoter activity of *fliC* in both induced and non-induced shaking flask cultivations.

Interestingly, when comparing growth curves of HMS174(DE3)LT7 we observed growth retardation upon induction. Since HMS174(DE3)LT7 exhibited class III promoter activity and overexpression of FliC has been shown to cause this effect [34], we were interested in determining if the deletion of both the *fliC* gene along with the *fliDST* operon would restore normal growth rates. First, we generated a HMS174(DE3) *ΔfliCDST* deletion strain to determine the general impact of this deletion on growth and subsequently compared results to HMS174(DE3)LT7 and HMS174(DE3)LT7 *ΔfliCDST* under non-induced and induced conditions. All three strains exhibited similar growth under non-induced conditions. In contrast to HMS174(DE3) *ΔfliCDST*, both HMS174(DE3)LT7 and HMS174(DE3)LT7 *ΔfliCDST* showed growth retardation upon induction with IPTG (Figure S2).

To rule out that this was a result of cell death, viability of the strains HMS174(DE3)LT7 and HMS174(DE3)LT7 *ΔfliCDST* were compared to HMS174(DE3) via FACS. However, no considerable differences in the cell viability of the respective strains could be detected (Figure S2).

### Impact of host strain modifications on type III secretion of FlgM

The high homogeneity of HMS174(DE3)LT7 cultures regarding the class III promoter activity indicated that this strain was ideal to investigate host strain modifications and their impact on FlgM as a secretion moiety. Several strains listed in Table 1 were tested regarding the influence of their respective modifications on FlgM secretion efficiency.

**Table 1:** Used host strains.

Strain name^a^	Genotype	FlgM found in supernatant	Reference
HMS174(DE3)	F- recA1, hsdR,(rK12- mK12+) (DE3), (Rif R)	+	Novagen
HMS174(DE3) Δ*fliCDST*	F- recA1, hsdR,(rK12- mK12+) (DE3), (Rif R), *ΔfliCDST*	−/+	This study
HMS174(DE3)Δ*insAB,* araBAD-*flhDC*	F- recA1, hsdR,(rK12- mK12+) (DE3), (Rif R), *ΔinsAB, araBAD-flhDC*	-	This study
HMS174(DE3) Δ*insAB,* araBAD*-flhDC*, Δ*fliT*	F- recA1, hsdR,(rK12- mK12+) (DE3), (Rif R), *ΔinsAB, araBAD-flhDC,ΔfliT*	-	This study
HMS174(DE3) Δ*insAB,* araBAD*-flhDC*, Δ*fliT, ΔflgKL*	F- recA1, hsdR,(rK12- mK12+) (DE3), (Rif R), *ΔinsAB,* araBAD-*flhDC, ΔfliT,ΔflgKL*	-	This study
HMS174(DE3)L^a^	F- recA1, hsdR,(rK12- mK12+) (DE3), (Rif R), *ΔinsAB,* lacUV5*-flhDC*	-	This study
HMS174(DE3)LL^a^	F- recA1, hsdR,(rK12- mK12+) (DE3), (Rif R), *ΔinsAB,* lacUV5*-flhDC, att-* lacUV5*-flhDC-6His*	-	This study
HMS174(DE3)T7*-flhDC*	F- recA1, hsdR,(rK12- mK12+) (DE3), (Rif R), *ΔinsAB,* T7*-flhDC*	-	This study
HMS174(DE3)LT7^a^	F- recA1, hsdR,(rK12- mK12+) (DE3), (Rif R), *ΔinsAB,* lacUV5*-flhDC, att7-*T7*-flhDC-*6His	+	This study
HMS174(DE3) LT7, *ΔfliCDST* a	F- recA1, hsdR,(rK12- mK12+) (DE3), (Rif R), *ΔinsAB,* lacUV5*-flhDC, att7-*T7*-flhDC*-6His, *ΔfliCDST*	+	This study

a These names are used to describe each mutant strain in Results and Discussion.

The late class III FliC and FliD proteins are considered to be direct FlgM secretion competitors [15]. Since we could demonstrate class III promoter activity and assembly of the final filament in HMS174(DE3)LT7 (Figure 6), the late class III *fliC* gene and the *fliDST* operon were deleted, which was expected to increase FlgM secretion.

Densitometric quantification of FlgM found in the supernatant of normalized cell cultivations revealed twice the secretion efficiency of HMS174(DE3)LT7 when compared to the parental HMS174(DE3). This may be attributed to a higher fraction of HMS174(DE3)LT7 cells assembling a functional hook basal body to secrete FlgM. However, the depletion of the *fliCDST* genes led to reduced relative FlgM levels in the supernatant (Figure S3).

## Discussion

Using recombinantly expressed FlgM as a secretion moiety resulted in the isolation of the first *E. coli* mutant strain HMS174(DE3)LT7 exhibiting full flagellar morphogenesis upon addition of the inducer IPTG. More detailed investigations of the respective strain revealed high cell homogeneity regarding *fliC* promoter activity upon induction in shaking flasks.

Furthermore, we observed growth retardation upon induction with IPTG, even though no significant differences in cell viability could be detected. High FliC expression rates have previously been shown to result in slower growth rates [34]. Hence, we performed a deletion of the flagellar filament gene *fliC* and the operon *fliDST* assuming that the metabolic burden of the filament assembly led to the observed growth retardation. However, the deletion of the filament genes alone did not abolish this effect. We propose that proliferation upon induction proceeded until sufficient levels of the master operon within each single cell resulted in flagellar assembly. Further investigations will be necessary to determine if the observed growth retardation of the generated mutant strains is indeed a result of the metabolic burden due to the strong induction of the master operon or a direct connection between flagellar morphogenesis and cell proliferation, and therefore remains controversial [38], [39].

The establishment of an inducible type III system for the secretion of heterologous proteins to the supernatant in *E. coli* would be very appealing due to protection against cellular proteases and simplified downstream processing [16], [17]. Recent work showed the efficient secretion of a neuropeptide fused to FlgM in *Salmonella* [15]. We also observed high purity secretion of FlgM fusion peptides to the supernatant. This could be especially interesting for antimicrobial fusion peptides. As FliA was previously shown to bind at the C-terminus of FlgM, retaining it in a secretion competent state, this binding may prevent premature folding of toxic fusion peptides and effects on bacterial host cells [12], [15]. Plasmid-encoded overexpression of FlgM fusion proteins could lead to an imbalance of the stoichiometric equilibrium between cytoplasmic FlgM molecules and its binding partner, the chaperon FliA. As a result, premature folding of FlgM fusion peptides may lead to a partial secretion incompetency, lowering overall secretion efficiencies. Optimization of this imbalance could potentially further increase secretion rates of recombinantly overexpressed FlgM fusion targets.

The late class III proteins FliC and FliD are considered to be direct secretion competitors to FlgM [15]. However, combined deletion of *fliC* and the operon *fliDST* resulted in a slight decrease of the overall secretion efficiency in the inducible strain. The decrease in FlgM secretion was even more prominent in the non-inducible strain HMS174(DE3) *ΔfliCDST*. This result was rather unexpected as deletions of *fliC* and *fliD* have been shown to be beneficial on secretion efficiencies of heterologous proteins [15], [21], and therefore suggested additional regulation mechanisms involved in the secretion of FlgM.

### Conclusion

The complexity of the flagellar system represents a severe hurdle in the establishment of a secretion system for heterologous proteins. We propose that using FlgM as a tool to evaluate the impact of gene modifications in the novel, inducible HMS174(DE3)LT7 *E. coli* strain may contribute to significant improvements in systematic engineering of an inducible type III secretion system.

## Methods

All experiments were performed with Milli-Q ultrapure water (Millipore purification system). *E. coli* cells were cultivated in TY growth medium supplemented with 50 µg/ml Kanamycin and/or 100 µg/ml Ampicillin depending on the used plasmid. For recombinant plasmid isolation the *E. coli* DH5α strain was used, whereas protein expression was performed in HMS174(DE3) and derived HMS174(DE3) mutant strains. Restriction enzymes, GoTaq® DNA polymerase, including the PCR buffer, were obtained from Promega. Molecular mass standard used for SDS-PAGE was purchased from Fermentas and Biorad. Rapid DNA ligation kit, Pfu DNA polymerase and 10× MgSO_4_-PCR buffer were obtained from Fermentas. Tris-Glycine gels were purchased from Invitrogen and Biorad. Protran BA 83 nitrocellulose membrane was obtained from Whatman. The monoclonal Anti-His antibody was obtained from Clontech. Syringe filters (pore size 0.45 µm) were from Sartorius.

### Cultivation of HMS174(DE3) strains for the flagellar secretion system

TY-medium conditioned with the appropriate antibiotic was inoculated with a single colony and incubated overnight at 37°C/225 rpm. This culture was diluted 1∶20 and incubated at 37°C/225 rpm until OD600: 0.5 for induction with 1 mM IPTG. Before induction 500 µl of the cell pellet and 1000 µl of the supernatant were collected. The cells were cultivated for 2 h–4 h at room temperature (RT)/225 rpm or 37°C/225 rpm. After cultivation the optical density was determined and the samples were normalized to OD 600: 0.5. 1000 µl samples of the supernatant were collected independent of the absorbance values. After incubation, the cells were harvested by centrifugation 15000 rpm/15 min (table top microcentrifuge) at room temperature. Supernatant was filtrated with Syringe filters (pore size 0.45 µm) from Sartorius and precipitated with TCA. The cell pellet and the TCA precipitated supernatant samples were resuspended in 50 µl SDS loading dye. For SDS-PAGE 10 µl and for immuno blot 2 µl of each sample was applied on SDS-PAGE.

### Small-scale cultivation

TY-medium was inoculated with a single colony and incubated overnight at 37°C/225 rpm. This culture was diluted 1∶20 and 6 ml were incubated at 37°C/225 rpm until OD 600: 0.5. Subsequently, the culture was split in 3 ml cultures with and without induction with 1 mM IPTG. The cells were cultivated for 3 h at 37°C/225 rpm. OD 600 was measured every 30 min.

### Scanning Electron Microscopy (SEM)

After cultivation for 90 min the bacterial suspensions were transferred on to Poly-L-lysine (Sigma-Aldrich) coated coverslips and incubated for 5 minutes on ice. Subsequently, the bacterial suspension was sucked off and immediately fixed with 2.5% glutaraldehyde (BioChemika Fluka) in 0.1 mol/l) in phosphate buffer (pH 7.4). After a brief wash in phosphate buffer, followed by postfixation for 1 h with 1% aqueous osmium tetroxide (ReagentPlus; Sigma-Aldrich), samples were gradually dehydrated with ethanol. After critical point drying (CPD 030, Bal-Tec), specimens were mounted on aluminum stubs with double-sided adhesive tape, sputter-coated with 10-nm Au Δ Pd (Bal-Tec) and examined with a field emission scanning electron microscope (Gemini 982; Zeiss, Goettingen, Germany).

### Fluorescence Activated Cell Sorting

After cultivation for 90 min the samples were normalized to OD 600: 1.0 with TY-media and subjected to Fluorescence Activated Cell Sorting. Cell viability was determined using Propidium iodide. Triplicates of all samples were subjected to Fluorescence Activated Cell Sorting.

### Gene Deletion/Insertion

For the disruption of genes in *E. coli* HMS174(DE3), the Quick & Easy *E. coli* Gene Deletion Kit (Genebridges) was used. The kit was used according to the manufactures recommendations. The genome modifications were verified via colony PCR and subsequent sequencing of the PCR product. For gene insertion the FRT-cassette was sub cloned into a plasmid and the gene of interest was fused 3′ to the FRT cassette. Subsequently, this fusion construct was amplified via PCR and used similar to the manufactures recommendations for gene deletions. Removal of the selection marker by FLP/FLPe expression was performed according to the manufactures recommendations. The removal of the selection marker cassette was controlled via colony PCR and subsequent sequencing of the PCR product. All primers are given in table 2 (Supplementary data).

### Gene deletion

For the disruption of the *fliCDST* genes in *E. coli* HMS174(DE3) the FRT-PGK-gb2-neo-FRT cassette was amplified via PCR using the primer pair 18/19. All subsequent steps were performed according to the manual supplied by Genebridges. The deletion of the target genes and the removal of the selection marker cassette were controlled via colony PCR and subsequent sequencing of the PCR product using the primer pair 20/21. For the disruption of the gene *fliT* the FRT-PGK-gb2-neo-FRT cassette was amplified via PCR using the primer pair 52/53. All subsequent steps were performed according to the manual supplied by Genebridges. The deletion of the target genes and the removal of the selection marker cassette were controlled via colony PCR and subsequent sequencing of the PCR product using the primer pair 34/43.

### Gene insertion

For the integration of heterologous genes the FRT cassette supplied by Genebridges was amplified via PCR using primer 1/2 and sub cloned into the pet32 vector using the NotI/XhoI restriction sites. The resulting pet32-FRT-PGK-gb2-neo-FRT was used for subsequent cloning steps. For the integration of the lacUV5 promoter into the *E. coli* genome, the lacUV5 promoter was directly ligated into the XhoI digested pet32-FRT-PGK-gb2-neo-FRT vector using the primer pair 3/4. Subsequently, the resulting FRT-PGK-gb2-neo-FRT-lacUV5 cassette was amplified using the primer pair 5/6. The amplified insert was used for the integration of the lacUV5 promoter according to the manual provided by Genebridges. The removal of the selection marker cassette and integration of the target gene was controlled via colony PCR and subsequent sequencing of the PCR product using the primer pair 9/10. For the integration of FRT-PGK-gb2-neo-FRT-lacUV5-*flhDC* into the *E. coli* genome at the att7 integration site, the strain HMS174(DE3) FRT-PGK-gb2-neo-FRT-lacUV5-*flhDC* was used to amplify FRT-PGK-gb2-neo-FRT-lacUV5-*flhDC* using the primer pair 11/12. The PCR insert was sub cloned into the vector pet32 using SalI/NotI as restriction sites. Subsequently, the resulting FRT-PGK-gb2-neo-FRT-lacUV5-*flhDC-6His*-T7term cassette was amplified using the primer pair 13/14. All subsequent steps were performed as recommended by Genebridges. The removal of the selection marker cassette and the integration of the target gene were controlled via colony PCR and subsequent sequencing of the PCR product using the primers 15/16. For the integration of the T7 promoter into the *E. coli* genome, the T7 promoter was directly ligated into the XhoI digested pet32-FRT-PGK-gb2-neo-FRT vector using the primer pair 7/8. Subsequently, the resulting construct FRT-PGK-gb2-neo-FRT-T7 was amplified using the primer pair 5/17 for the generation of the strain HMS174(DE3) T7-*flhDC*. All subsequent steps were performed as recommended by Genebridges. The removal of the selection marker cassette and integration of the target gene was controlled via colony PCR and subsequent sequencing PCR product using the primers 9/10. The T7 promoter at the att7 site was integrated using the primer pair for 13/17 amplifying FRT-PGK-gb2-neo-FRT-T7 cassette. All subsequent steps were performed as recommended by Genebridges. The removal of the selection marker cassette and integration of the target gene was controlled via colony PCR and subsequent sequencing of the PCR product using the primer pair 15/16.

For the integration of the araBAD promoter into the *E. coli* genome, the araBAD promoter was directly ligated into the XhoI digested pet32-FRT-PGK-gb2-neo-FRT vector using the primer pair 47/48. Subsequently, the resulting FRT-PGK-gb2-neo-FRT-araBAD cassette was amplified using the primer pair 5/49. All subsequent steps were performed as recommended by Genebridges. The removal of the selection marker cassette and integration of the target gene was controlled via colony PCR and subsequent sequencing of the PCR product using the primer pair 9/10.

### Construction of the expression plasmid

#### pET30-lacUV5-flgM-6His

Initially, the pET30 vector was digested with SphI/Xba. Mixing the primer pair 22/23 and subsequent ligation in the pET30 vector resulted in the pET30-lacUV5 vector. The insert *flgM* was amplified using the primer pair 24/25 and cloning into the prepared vector pET30-lacUV5. The resulting p30-lacUV5-*flgM-6His* construct was verified via sequencing using the primer pair 26/27.

#### p5′UTR-fliC20-GFPmut-Flag-Linker-StrepII-3′UTR

The 5′untranslated region (UTR) of the gene *fliC* was cloned into the pET30 vector to replace the T7 promoter. Within this UTR, the promoter of the *fliC* gene is encoded [21]. First, the pET30 vector was digested with SphI and NdeI to remove the T7 promoter of pET30. The 5′UTR insert was generated via gene assembly using the primers 28–33 and subsequent cloning into the prepared vector. Afterwards, the newly generated construct was confirmed via sequencing using the primer 26. The p5′UTR vector was subsequently prepared for the insertion of the 3′UTR insert. The 3′UTR was amplified via PCR with the primer pair 35/36 from the strain DH5α. The resulting construct p5′UTR-3′UTR was verified via sequencing with the primer pair 26/27. This construct was prepared for the insertion of *fliC20*. The primer pair 37/38 was directly ligated into the vector resulting in the p5′UTR-*fliC20*-*Flag-Linker-StrepII*-3′UTR construct. For the final p5′UTR-*fliC20-GFPmut-Flag-Linker-StrepII-3*′UTR construct *GFPmut* was amplified using the primer pair 39/40. After digestion of the insert and subsequent ligation, the construct was verified via sequencing using the primer pair 26/27.

#### pET32-lacUV5-flhDC

The operon *flhDC* was amplified using the host strain HMS174(DE3) as template with the primer pair 41/42 and cloned into the prepared vector pET32-lacUV5. The resulting pET32-lacUV5-*flhDC* construct was verified via sequencing using the primer pair 26/27.

#### pET30-lacUV5-flgM-SOD, pET30-lacUV5-flgM-GFPmut-6H

The insert *SOD* was amplified using the primer pair 45/46 and cloned into the prepared vector pET30-lacUV5. The resulting pET32-lacUV5-*flgM-SOD* construct was verified via sequencing using the primer pair 26/27. GFPmut was amplified using the primer pair 50/51 and cloned into the prepared vector pET30-lacUV5. The resulting constructs were verified via sequencing using the primer pair 26/27.

### RNA isolation

Samples were collected after cultivation and OD600 was determined to isolate the RNA with TRI Reagent™ according to the manual provided by Sigma.

DNase Reaction: 1 µg of the obtained RNA samples were subjected to DNase digestion to eliminate co purified DNA according the manual provided by Fermentas.

### Reverse Transcription Reaction

Reverse Transcription Reaction for the RT-PCR experiment of plasmid-encoded *flhDC* was performed according the manual provided Fermentas Revert Aid™Minus First Strand cDNA Synthesis Kit using the primer 41. Subsequent PCR Reaction was performed using the primer pair 41/44.

### Quantitative Real-Time PCR Reaction

Total RNA was isolated using TRIzol reagent (Invitrogen). After chloroform extraction and isopropanol precipitation, resulting RNA yield and purity was determined with a spectrophotometer. Subsequent cDNA synthesis was done with SuperscriptII reverse transcriptase (Invitrogen) according to the manual from 5 µg of RNA using specific primer for rpoD (loading control) and flhD. Quantitative real-time PCR was performed using Sybrgreen (Company), Pfu polymerase (Fermentas) and the following gene specific primer 54/55 for rpoD and 56/57 for flhD. The amplification of cDNA was performed by Opticon Real-Time PCR System (MJ Research). Quantification of flhD was done applying the comparative ΔΔCt method using rpoD for normalization. Resulting data were analyzed via 2-tailed type 2 Student's *t*-test.

### Anti-His tag immuno blot

Blotting of the proteins samples was performed according to the manufacturers recommendations (Invitrogen). The blotting chamber Invitrogen XCell-II Blot module was filled with transfer buffer (0.3% Tris, 1.5% glycine, 20% methanol, 0.02% SDS). The protein samples were blotted for 90 min at 35 Volt. The Anti-His immuno blot was performed as recommended in the manual for monoclonal Anti-His antibodies provided from Clontech.

## Supporting Information

Figure S1 **FlgM as a sensor protein for the development of an inducible type III secretion system.** Plasmid-encoded overexpression of the FlgM protein facilitated a straightforward detection of mutant strains efficiently secreting the protein across the macromolecular flagellar structure to the supernatant via SDS-PAGE. Whereas in HMS174(DE3) FlgM secretion was observed the generated mutant strains HMS174(DE3)*ΔinsAB* lacUV5-*flhDC* (HMS174(DE3)L), HMS174(DE3)*ΔinsAB* T7-*flhDC*, HMS174(DE3)Δ*insAB* araBAD-*flhDC ΔfliT* lacked the ability to secrete this protein to the supernatant. (**A**) SDS-PAGE, (**B**) Anti 6His-tag immuno blot, M Fermentas PageRuler Prestained, -I whole cell sample without induction of recombinant protein expression, +I whole cell samples with induction of recombinant protein expression, C cytoplasmic fraction, Sn supernatant;
(TIF)

Figure S2 **Analysis of growth and cell viability.** (A) Cell growth. Three clones of HMS174(DE3) ΔfliCDST, (B) HMS174(DE3)LT7 and (C) HMS174(DE3)LT7 ΔfliCDST were cultivated for 3 h with and without addition of the inducer IPTG [1 mM]. The optical density was measured every 30 min to determine differences in cell growth. (D) Cell viability. Samples of HMS174(DE3), HMS174(DE3)LT7 and HMS174(DE3)LT7ΔfliCDST cultivations +/− IPTG were normalized to OD600: 1.0 after incubation for 90 min and subjected to FACS analysis using Propidiumiodide to determine cell viability.
(TIF)

Figure S3 **Comparison of the FlgM secretion efficiency.** Plasmid-encoded FlgM-6His was overexpressed in the given host strains HMS174(DE3), HMS174(DE3)ΔfliCDST, HMS174(DE3)LT7 and HMS174(DE3)LT7ΔfliCDST. Upon induction with IPTG and incubation for 2 h/37°C/225 rpm the cultures were subsequently normalized to OD600: 1.0 to improve comparability. Normalized protein samples derived from the supernatant were precipitated and subjected to SDS-PAGE. Subsequently, three samples of each expression were densitometrically quantified. The highest secretion value was considered as 100% secretion efficiency.
(TIF)

Figure S4 **Evaluation of FlgM as a secretion moiety in HMS174(DE3).** The human Superoxide Dismutase (SOD) and GFPmut3.1-6His genes were fused 3′ to the flgM gene. These fusion constructs were recombinantly expressed for 4 h/RT/225 rpm in HMS174(DE3). Samples were subjected to SDS-PAGE. FlgM mediates the secretion of SOD to the supernatant whereas GFPmut3.1-6His fused to FlgM is not found in the supernatant. (A) Expression of FlgM-SOD in HMS174(DE3), (B) Expression of FlgM-GFP3.1mut-6H in HMS174(DE3) M Biorad Precision Plus Protein Dual Color Standard, -I whole cell sample without induction of recombinant protein expression, +I whole cell samples with induction of recombinant protein expression, C cytoplasmic fraction, Sn supernatant.
(TIF)

Table S1 **Primers used in this study.**
(DOCX)

Sequences S1 **Sequence of the plasmid encoded master operon** ***flhDC*** **under the control of the lacUV5 promoter.** Sequences of plasmid encoded *flgM* fusion constructs under the control of the lacUV5 promoter.
(DOCX)
