# Supplementary figures and images for: FlgM as a Secretion Moiety for the Development of an Inducible Type III Secretion System
Source: PLoS One. 2013 Mar 12;8(3):e59034. doi: 10.1371/journal.pone.0059034 (PMC3595227; doi:10.1371/journal.pone.0059034)

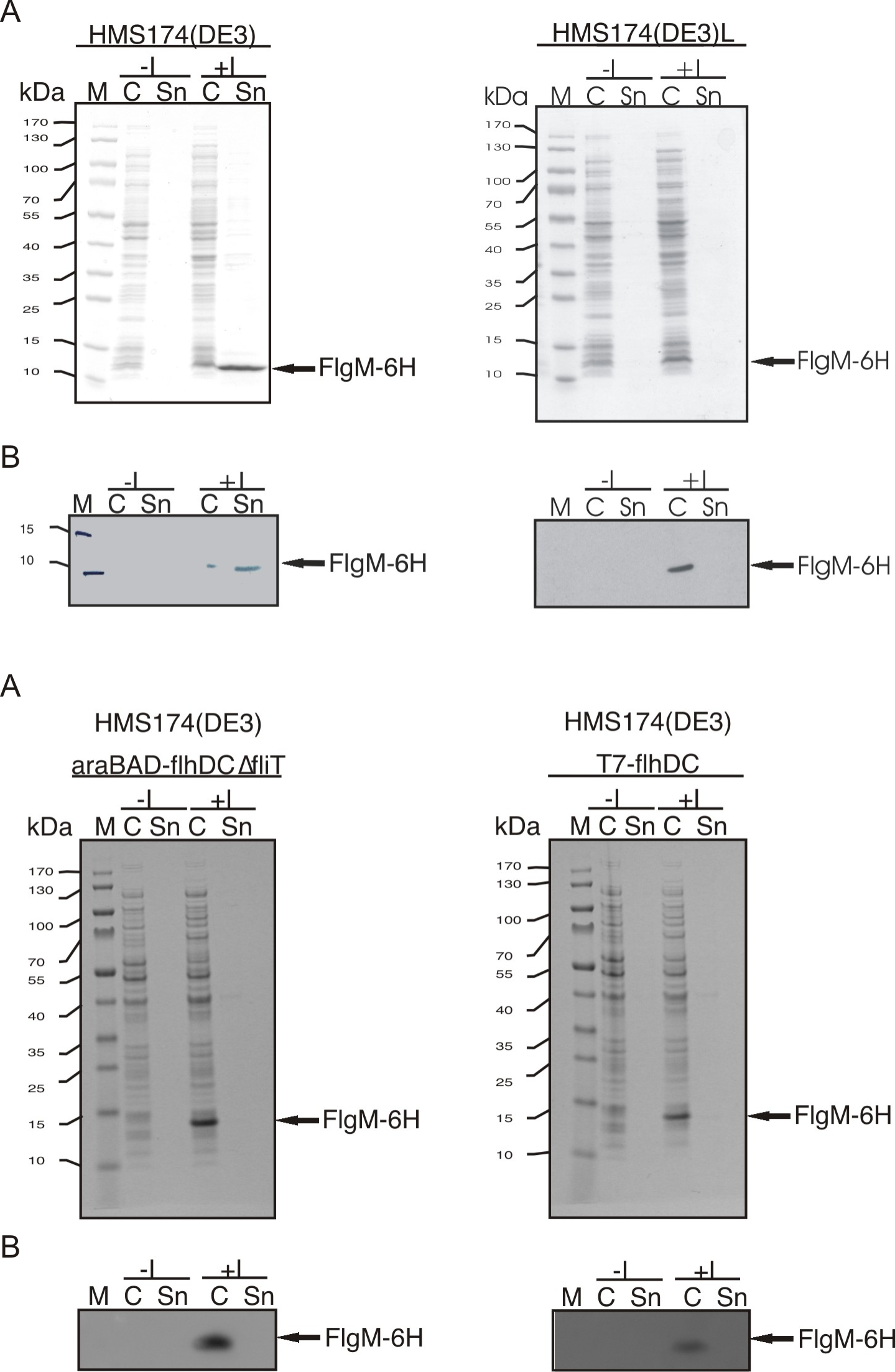

Supplement: Figure S1 — FlgM as a sensor protein for the development of an inducible type III secretion system. Plasmid-encoded overexpression of the FlgM protein facilitated a straightforward detection of mutant strains efficiently secreting the protein across the macromolecular flagellar structure to the supernatant via SDS-PAGE. Whereas in HMS174(DE3) FlgM secretion was observed the generated mutant strains HMS174(DE3)ΔinsAB lacUV5-flhDC (HMS174(DE3)L), HMS174(DE3)ΔinsAB T7-flhDC, HMS174(DE3)ΔinsAB araBAD-flhDC ΔfliT lacked the ability to secrete this protein to the supernatant. (A) SDS-PAGE, (B) Anti 6His-tag immuno blot, M Fermentas PageRuler Prestained, -I whole cell sample without induction of recombinant protein expression, +I whole cell samples with induction of recombinant protein expression, C cytoplasmic fraction, Sn supernatant; (TIF) [file pone.0059034.s001.tif]

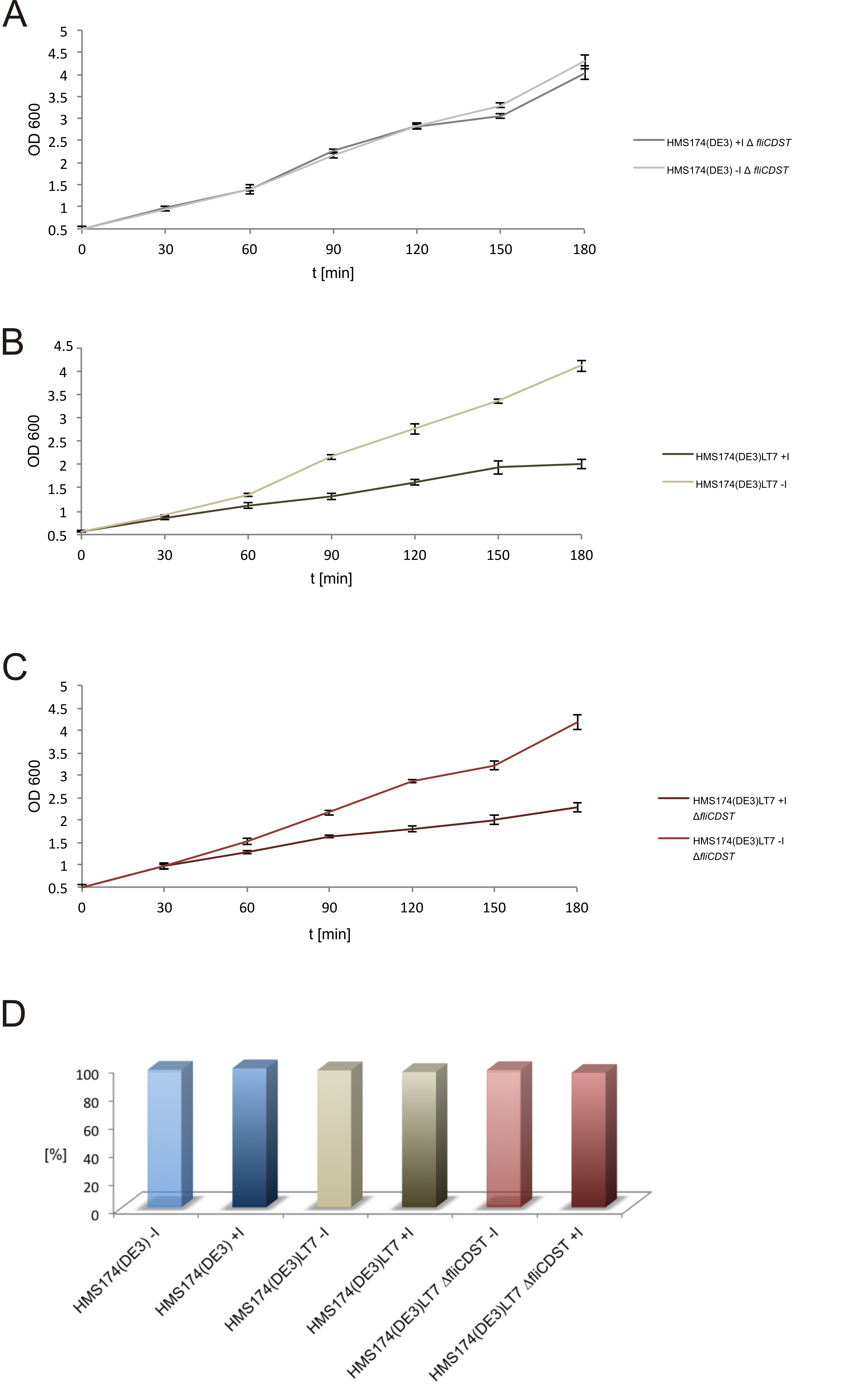

Supplement: Figure S2 — Analysis of growth and cell viability. (A) Cell growth. Three clones of HMS174(DE3) ΔfliCDST, (B) HMS174(DE3)LT7 and (C) HMS174(DE3)LT7 ΔfliCDST were cultivated for 3 h with and without addition of the inducer IPTG [1 mM]. The optical density was measured every 30 min to determine differences in cell growth. (D) Cell viability. Samples of HMS174(DE3), HMS174(DE3)LT7 and HMS174(DE3)LT7ΔfliCDST cultivations +/− IPTG were normalized to OD600: 1.0 after incubation for 90 min and subjected to FACS analysis using Propidiumiodide to determine cell viability. (TIF) [file pone.0059034.s002.tif]

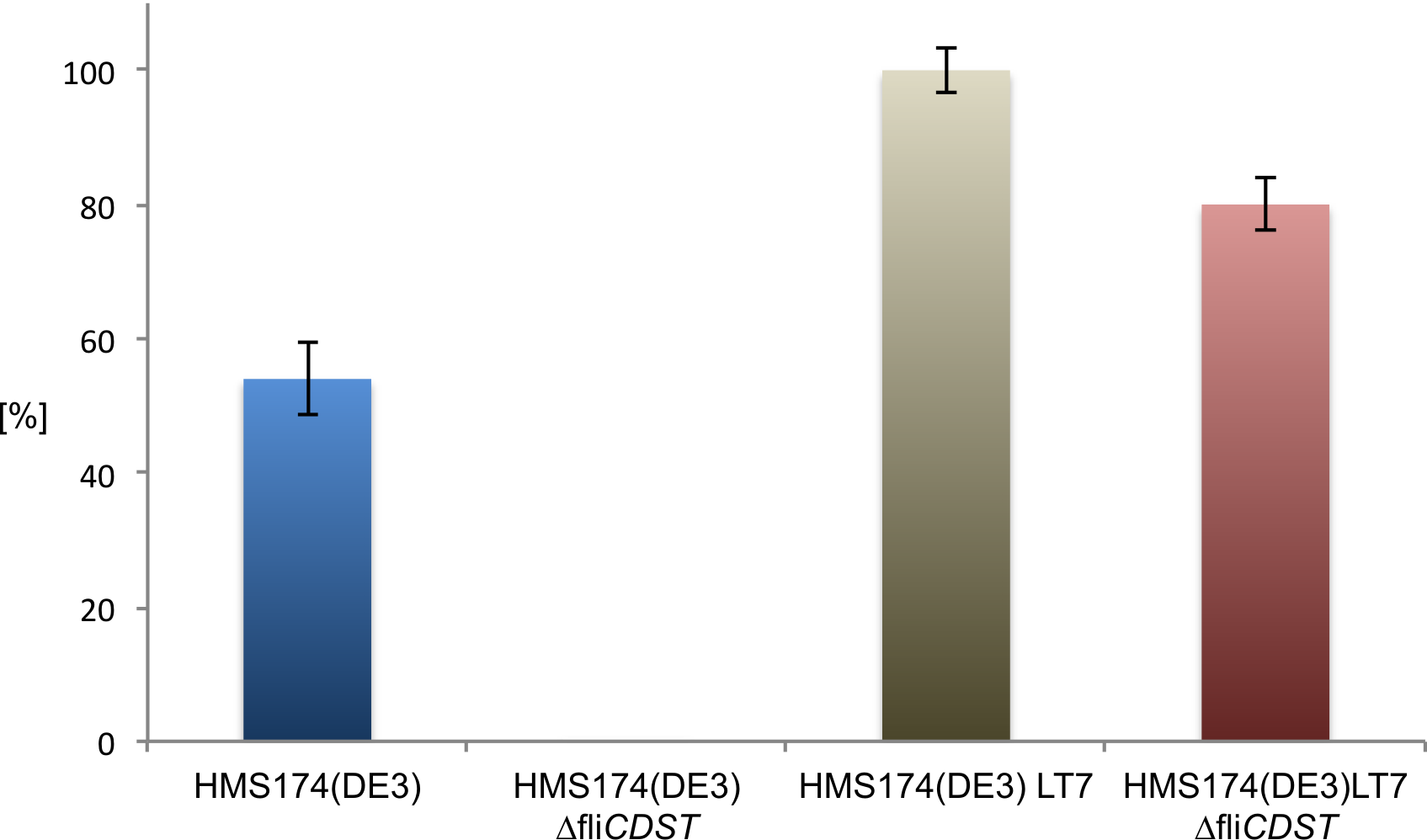

Supplement: Figure S3 — Comparison of the FlgM secretion efficiency. Plasmid-encoded FlgM-6His was overexpressed in the given host strains HMS174(DE3), HMS174(DE3)ΔfliCDST, HMS174(DE3)LT7 and HMS174(DE3)LT7ΔfliCDST. Upon induction with IPTG and incubation for 2 h/37°C/225 rpm the cultures were subsequently normalized to OD600: 1.0 to improve comparability. Normalized protein samples derived from the supernatant were precipitated and subjected to SDS-PAGE. Subsequently, three samples of each expression were densitometrically quantified. The highest secretion value was considered as 100% secretion efficiency. (TIF) [file pone.0059034.s003.tif]

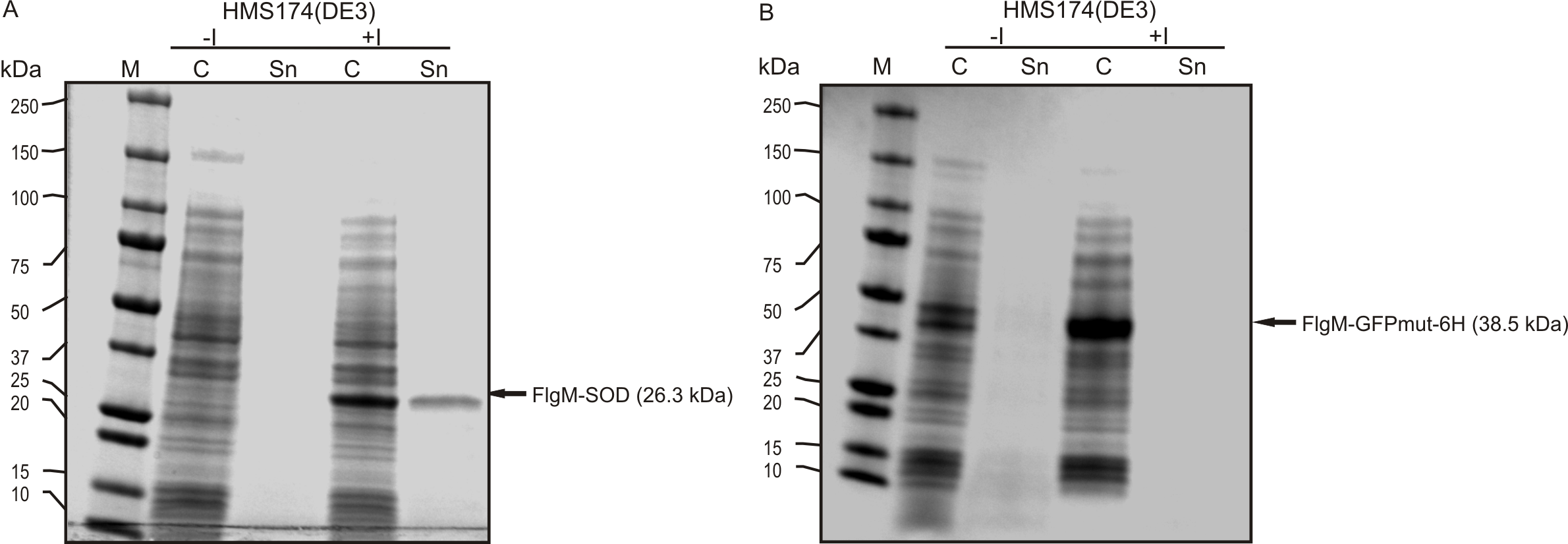

Supplement: Figure S4 — Evaluation of FlgM as a secretion moiety in HMS174(DE3). The human Superoxide Dismutase (SOD) and GFPmut3.1-6His genes were fused 3′ to the flgM gene. These fusion constructs were recombinantly expressed for 4 h/RT/225 rpm in HMS174(DE3). Samples were subjected to SDS-PAGE. FlgM mediates the secretion of SOD to the supernatant whereas GFPmut3.1-6His fused to FlgM is not found in the supernatant. (A) Expression of FlgM-SOD in HMS174(DE3), (B) Expression of FlgM-GFP3.1mut-6H in HMS174(DE3) M Biorad Precision Plus Protein Dual Color Standard, -I whole cell sample without induction of recombinant protein expression, +I whole cell samples with induction of recombinant protein expression, C cytoplasmic fraction, Sn supernatant. (TIF) [file pone.0059034.s004.tif]
