# Supplementary material for: FlgM as a Secretion Moiety for the Development of an Inducible Type III Secretion System
Source: PLoS One. 2013 Mar 12;8(3):e59034. doi: 10.1371/journal.pone.0059034 (PMC3595227; doi:10.1371/journal.pone.0059034)
Supplement: Table S1 — Primers used in this study. (DOCX) [file pone.0059034.s005.docx]

**Table S1: Primers used in this study**

| 1 | FRT-PGK-gb2- neo-  FRT Not F | AATTAACCCTCACTAAAGGGCG |  |
| --- | --- | --- | --- |
| 2 | FRT-PGK-gb2- neo-FRT Xho R | TAATACGACTCACTATAGGGCTC |  |
| 3 | pet32-FRT-lacUV5 RBS XhoF | 5´-**P** TCGAGCCAGGCTTTACACTTTATGCTTCCGGCTCGTATAATGTGTGGAATTGTGAGCGGATAACAATTTCACACAGGAAACAG C |  |
| 4 | pet32-FRT-lacUV5 RBS XhoR | 5´-**P** TCGAG CTGTTTCCTGTGTGAAATTGTTATCCGCTCACAATTCCACACATTATACGAGCCGGAAGCATAAAGTGTAAAGCCTGG C |  |
| 5 | knock out insAB F | TTTTGACTGTGCGCAACATCCCATTTCGATTATTCCTGTTTCATTTTTGCTTGCTAGCGTAGCGAAAAACTTTTTAACAGATTGGCGGCCGCGAAGTTCCTATTC |  |
| 6 | knock in lacUV5 R | CTGAACAATCAAACGCTGTGCAAGTAGTAAATATGACAAGTTGATGTCATAAATGTGTTTCAGCAACTCGGAGGTATGCATCTCGAGCTGTTTCCTGTG |  |
| 7 | T7 -RBS-Prom Xho F | 5´-**P** TCGAGTTAATACGACTCACTATAGGGGAATTGTGAGCGGATAACAATTCCCCTCTAGAAATAATTTTGTTTAACTTTAAGAAGGAGATC |  |
| 8 | T7-RBS-PromXhoR | 5´-**P** TCGAGATCTCCTTCTTAAAGTTAAACAAAATTATTTCTAGAGGGGAATTGTTATCCGCTCACAATTCCCCTATAGTGAGTCGTATTAAC |  |
| 9 | flhDC prom. control F | AAACAAGTGGTTAATATATG |  |
| 10 | flhDC prom. control R | GCTGTCAAAACGGAAGTGAC |  |
| 11 | FRT-PGK-gb2-neo-  Frt SalI F | AAAAGTCGACCCGCGAAGTTCCTATTCTCTAGAAAG |  |
| 12 | flhDC NotI R | TTTGCGGCCGCAACAGCCTGTACTCTCTGTTCATC |  |
| 13 | att7 prom.-flhDC  knock in F | TAAATGGATGCCCTGCGTAAGCGGGGCATTTTTCTTCCTGTTATGTTTTTAATCAAACATCCTGCCAACTCCATGTGAC GAGCTCCGTCGACCCGCGAAG |  |
| 14 | att7 prom.-  flhDC knock in R | AATAAGCGTTGATATTCAGTCAATTACAAACATTAATAACGAAGAGATGACAGAAAAATTTTCATTCTGTGACAGAGAAAA CCGGATATAGTTCCTCCTTTCAG |  |
| 15 | att7 prom.-flhDC  knock in Seq F | GAGATGCCGCATGTGGAA |  |
| 16 | att7 prom.-flhDC  knock in Seq R | AAATAGGACAAACAGGTGACAG |  |
| 17 | knock in T7 Prom R | AACAATCAAACGCTGTGCAAGTAGTAAATATGACAAGTTGATGTCATAAATGTGTTTCAGCAACTCGGAGGTATGCATCTCGAGATCTCCTTCTTAAAG |  |
| 18 | fliCDST knock out F | TCAACTTGTAGGCCTGATAAGCGCAGCGCATCAGGCAATTTGGCGTTGCCGTCAGTCTCAGTTAATCAGGTTACAACGAAATTAACCCTCACTAAAGGGCG |  |
| 19 | fliCDST knock out R | TTCGACTCCATTCAAGGGGAACATTAGAAGCGTAGCCGTAATCGGATTATTCGCGAGCCATCGACTCATTCAGATTCATAATACGACTCACTATAGGGCTC |  |
| 20 | fliCDST control primer F | TCAACTTGTAGGCCTGATAAG |  |
| 21 | fliCDST control primer R | CCAGAGCTTACCGCCTTCCGGG |  |
| 22 | prom LacUV5 SpH F | 5´**P**-CCCAGGCTTTACACTTTATGCTTCCGGCTCGTATAATGTGTGGAATTGTGAGCGGATAACAATTT | |
| 23 | prom LacUV5 Xba R | 5´**P**-CTAGAAATTGTTATCCGCTCACAATTCCACACATTATACGAGCCGGAAGCATAAAGTGTAAAGCCTGGGCATG |  |
| 24 | pet30 FlgM NdeI F | TGACCATATGAGTATTGATCGCACTTCG | |
| 25 | pet30 FlgM Sal R | TTTGTCGACGTTACTCTGCAAGTCTTGCTG | |
| 26 | FliC seq 5´UTR F | AGCACCGCCGCCGCAAGGAAT | |
| 27 | T7 Term 21 R | GTTATGCTAGTTATTGCTCAG | |
| 28 | FliC SphI 5´UTR F | TTTGCATGCTAATAGCGGGAATAAG | |
| 29 | 5´UTR gensyn2 | ATTTTTTGTTAGTCGCCGAAATACTCTTTTCTCTGCCCCTTATTCCCGCTATTAGCATGC | |
| 30 | 5´UTR gensyn3 | GTATTTCGGCGACTAACAAAAAATGGCTGTTTTTGAAAAAAATTCTAAAGGTTGTTTTAC | |
| 31 | 5´UTR gensyn4 | TCAATCGCCGTCAACCCTGTTATCGTCTGTCGTAAAACAACCTTTAGAATTTTTTTCAAA | |
| 32 | 5´UTR gensyn5 | AGGGTTGACGGCGATTGAGCCGACGGGTGGAAACCCAATACGTAATCAACGACTTGCAAT | |
| 33 | FliC NdeI 5´UTR R | ACGGTACATATGATTCGTTATCCTATATTGCAAGTCGTTGATTACGTAT | |
| 34 | FliT control primer F | TATATGGTCAGGCGCTTGCTG | |
| 35 | FliC NotI 3´UTR F | AGCGGCCGCTCGTTGTAACCTGATTAA | |
| 36 | FliC XhoI 3´UTR | AATCTCGAGAATTCACGATAAACAGCC | |
| 37 | pET30 FliC20 Spe R | 5´**P**-CTAGTCTTGTTGATATTATTT TGAGTGATCAGCGAGAGGCTGTTGGTATTAATGACTTGTGCCA | |
| 38 | pET30 FliC20 Nde F | 5´**P**-TATGGCACAAGTCATTAATACCAACAGCCTCTCGCTGATCACTCAAAATAATATCAACAAGA | |
| 39 | pET30 GFPmut Spe R | ACTAGTTTTGTATAGTTCATCCATGCC | |
| 40 | pET30 GFPmut Spe F | ACTAGTAAAGGAGAAGAACTTTTCACTG | |
| 41 | flhDC Xba/ F | ACAGTCTAGAATGCATACCTCCGAGTTG | |
| 42 | flhDC Bam/Xho R | CCTTACTCGAGGGATCCTTAAACAGCCTGTACTCTCTG | |
| 43 | FliT control primer R | CCAGAGCTTACCGCCTTCCGGG | |
| 44 | flhDC intern RT | ACGCCATTACACAAACCGG | |
| 45 | FlgM SOD Sal F | TTACGTCGACGCAACAAAGGCGCTGTGC | |
| 46 | SOD Sal R | AAATGTCGACTTATTGGGCGATCCCAATTAC | |
| 47 | araBAD Xho F | TTGCTCGAGAAGAAACCAATTGTCCATATTGC | |
| 48 | araBAD Xho R | AAACCTCGAGCCCATGGTTAATTCCTCCTGTTAGCCCAAAAAACGGGTATGGAGAAAC | |
| 49 | FlhDC Xho  AraBAD R | AACAATCAAACGCTGTGCAAGTAGTAAATATGACAAGTTGATGTCATAAATGTGTTTCAGCAACTCGGAGGTATGCATCTCGAGCCCATGGTTAATTCC | |
| 50 | \| GFP mut3.1 Sal F \| \| --- \| \|  \| | AAAGTCGACAGCAAAGGCGAAGAACTG | |
| 51 | GFP mut3.1 Not R | TTTGCGGCCGCTTTATACAGTTCATCCATG | |
| 52 | FliT knock out F | GTGGAAGCATTAATGCGCAATATTGCCGATGCCTGGAAAGAGTCGTTACTCTCCCCTTCTTTGATTCAGGACCCAGTCTGAATTAACCCTCACTAAAGGGCG | |
| 53 | FliT knock out R | TTCGACTCCATTCAAGGGGAACATTAGAAGCGTAGCCGTAATCGGATTATTCGCGAGCCATCGACTCATTCAGATTCATAATACGACTCACTATAGGGCTC | |
| 54 | rpoD fwd | ATGGAGCAAAACCCGCAGTCAC | |
| 55 | rpoD rev | TCTTCCATCACCTGAATGCCCATG | |
| 56 | flhD fwd | ATGCATACCTCCGAGTTGCTG | |
| 57 | flhD rev | AACCATTTGCGGAAGAGTCAGTG | |

5´-P represents a 5´phosphate group modification
