# Supplementary material for: FlgM as a Secretion Moiety for the Development of an Inducible Type III Secretion System
Source: PLoS One. 2013 Mar 12;8(3):e59034. doi: 10.1371/journal.pone.0059034 (PMC3595227; doi:10.1371/journal.pone.0059034)
Supplement: Sequences S1 — Sequence of the plasmid encoded master operon flhDC under the control of the lacUV5 promoter. Sequences of plasmid encoded flgM fusion constructs under the control of the lacUV5 promoter. (DOCX) [file pone.0059034.s006.docx]

lacUV5-*flhDC* (pET32*)*:

CCAGGCTTTACACTTTATGCTTCCGGCTCGTATAATGTGTGGAATTGTGAGCGGATAACAATTTCACACAGGAAACAGTCTAGAGTGGGAATAATGCATACCTCCGAGTTGCTGAAACACATTTATGACATCAACTTGTCATATTTACTACTTGCACAGCGTTTGATTGTTCAGGACAAAGCGTCCGCTATGTTTCGTCTCGGCATAAATGAAGAAATGGCGACAACGTTAGCGGCACTGACTCTTCCGCAAATGGTTAAGCTGGCAGAAACCAATCAACTGGTTTGTCACTTCCGTTTTGACAGCCACCAGACGATTACTCAGTTGACGCAAGATTCCCGCGTTGACGATCTCCAGCAAATTCATACCGGCATCATGCTCTCAACACGCTTGCTGAATGATGTTAATCAGCCTGAAGAAGCGCTGCGCAAGAAAAGGGCCTGATCATGAGTGAAAAAAGCATTGTTCAGGAAGCGCGGGATATTCAGCTGGCAATGGAATTGATCACCCTGGGCGCTCGTTTGCAGATGCTGGAAAGCGAAACACAGTTAAGTCGCGGACGCCTGATAAAACTTTATAAAGAACTGCGCGGAAGCCCACCGCCGAAAGGCATGCTGCCATTCTCAACCGACTGGTTTATGACCTGGGAACAAAACGTTCATGCTTCGATGTTCTGTAATGCATGGCAGTTTTTACTGAAAACCGGTTTGTGTAATGGCGTCGATGCGGTGATCAAAGCCTACCGTTTATACCTTGAACAGTGCCCACAAGCAGAAGAAGGACCACTGCTGGCATTAACCCGTGCCTGGACATTGGTGCGGTTTGTTGAAAGTGGATTACTGCAACTTTCCAGCTGCAACTGCTGCGGCGGCAATTTTATTACCCACGCTCACCAGCCTGTTGGCAGCTTTGCCTGCAGCTTATGTCAACCGCCATCCCGGGCAGTAAAAAGACGTAAACTTTCCCAGAATCCTGCCGATATTATCCCACAACTGCTGGATGAACAGAGAGTACAGGCTGTTTAA

lacUV5-*flgM-6His* (pET30):

CCAGGCTTTACACTTTATGCTTCCGGCTCGTATAATGTGTGGAATTGTGAGCGGATAACAATTTCACACAGGAAACAGTCTAGAAATAATTTTGTTTAACTTTAAGAAGGAGATATACATATGAGTATTGATCGCACTTCGCCTCTGAAGCCTGTAAGCACCGTTCAACCGCGCGAAACCACTGACGCGCCGGTAACGAACAGCCGGGCGGCAAAAACAACCGCCTCCACCAGCACCAGTGTGACGTTAAGCGACGCGCAAGCAAAACTGATGCAACCCGGCAGCAGTGATATCAATCTTGAACGTGTCGAAGCGTTAAAACTGGCGATTCGTAACGGTGAACTAAAAATGGACACCGGCAAAATTGCCGATGCGCTGATCAACGAAGCGCAGCAAGACTTGCAGAGTAACGTCGACAAGCTTGCGGCCGCACTCGAGCACCACCACCACCACCACTGA

lacUV5*-flgM-SOD* (pET30):

CCAGGCTTTACACTTTATGCTTCCGGCTCGTATAATGTGTGGAATTGTGAGCGGATAACAATTTCACACAGGAAACAGTCTAGAAATAATTTTGTTTAACTTTAAGAAGGAGATATACATATGAGTATTGATCGCACTTCGCCTCTGAAGCCTGTAAGCACCGTTCAACCGCGCGAAACCACTGACGCGCCGGTAACGAACAGCCGGGCGGCAAAAACAACCGCCTCCACCAGCACCAGTGTGACGTTAAGCGACGCGCAAGCAAAACTGATGCAACCCGGCAGCAGTGATATCAATCTTGAACGTGTCGAAGCGTTAAAACTGGCGATTCGTAACGGTGAACTAAAAATGGACACCGGCAAAATTGCCGATGCGCTGATCAACGAAGCGCAGCAAGACTTGCAGAGTAACGTCGACGCAACAAAGGCCGTGTGCGTGCTGAAGGGCGACGGCCCAGTGCAGGGCATCATCAATTTCGAGCAGAAGGAAAGTAATGGACCAGTGAAGGTGTGGGGAAGCATTAAAGGACTGACTGAAGGCCTGCATGGATTCCATGTTCATGAGTTTGGAGATAATACGGCAGGCTGTACCAGTGCAGGTCCTCACTTTAATCCTCTATCCAGAAAACACGGTGGGCCAAAGGATGAAGAGAGGCATGTTGGAGACTTGGGCAATGTGACTGCTGACAAAGATGGTGTGGCCGATGTGTCTATTGAAGATTCTGTGATCTCACTCTCAGGAGACCATTGCATCATTGGCCGCACACTGGTGGTCCATGAAAAAGCAGATGACTTGGGCAAAGGTGGAAATGAAGAAAGTACAAAGACAGGAAACGCTGGAAGTCGTTTGGCTTGTGGTGTAATTGGGATCGCCCAATAG

lacUV5*-flgM-GFPmut3.1-6His* (pET30):

CCAGGCTTTACACTTTATGCTTCCGGCTCGTATAATGTGTGGAATTGTGAGCGGATAACAATTTCACACAGGAAACAGTCTAGAAATAATTTTGTTTAACTTTAAGAAGGAGATATACATATGAGTATTGATCGCACTTCGCCTCTGAAGCCTGTAAGCACCGTTCAACCGCGCGAAACCACTGACGCGCCGGTAACGAACAGCCGGGCGGCAAAAACAACCGCCTCCACCAGCACCAGTGTGACGTTAAGCGACGCGCAAGCAAAACTGATGCAACCCGGCAGCAGTGATATCAATCTTGAACGTGTCGAAGCGTTAAAACTGGCGATTCGTAACGGTGAACTAAAAATGGACACCGGCAAAATTGCCGATGCGCTGATCAACGAAGCGCAGCAAGACTTGCAGAGTAACGTCGACAGCAAAGGCGAAGAACTGTTTACCGGTGTGGTGCCGATTCTGGTGGAACTGGATGGCGATGTGAACGGTCATAAATTTAGCGTGAGCGGCGAAGGTGAAGGCGATGCGACCTATGGTAAACTGACCCTGAAATTTATTTGCACCACCGGCAAACTGCCGGTGCCGTGGCCGACCCTGGTGACCACCTTTGGTTATGGCGTGCAGTGCTTTGCGCGCTATCCGGATCACATGAAACAGCATGATTTTTTTAAAAGCGCGATGCCGGAAGGTTATGTGCAGGAACGCACCATTTTTTTTAAAGATGATGGCAACTATAAAACCCGCGCGGAAGTGAAATTTGAAGGTGATACCCTGGTGAACCGCATTGAACTGAAAGGCATTGATTTTAAAGAAGATGGTAACATTCTGGGCCATAAACTGGAATATAACTATAACAGCCATAACGTGTATATTATGGCGGATAAACAGAAAAACGGTATTAAAGTGAACTTTAAAATTCGCCATAACATTGAAGATGGCAGCGTGCAGCTGGCGGATCATTATCAGCAGAACACCCCGATTGGTGATGGCCCGGTGCTGCTGCCGGATAACCATTATCTGAGCACCCAGAGCGCGCTGAGCAAAGATCCGAACGAAAAACGCGATCACATGGTGCTGCTGGAATTTGTGACCGCGGCGGGTATTACGCATGGCATGGATGAACTGTATAAAGCGGCCGCACTCGAGCACCACCACCACCACCACTGA
